# Prediction of Associations between OMIM Diseases and MicroRNAs by Random Walk on OMIM Disease Similarity Network

**DOI:** 10.1155/2013/204658

**Published:** 2013-03-20

**Authors:** Hailin Chen, Zuping Zhang

**Affiliations:** ^1^School of Information Science and Engineering, Central South University, Changsha 410083, China; ^2^Department of Computer Science and Technology, Hunan University of Humanities, Science and Technology, Loudi 417000, China

## Abstract

Increasing evidence has revealed that microRNAs (miRNAs) play important roles in the development and progression of human diseases. However, efforts made to uncover OMIM disease-miRNA associations are lacking and the majority of diseases in the OMIM database are not associated with any miRNA. Therefore, there is a strong incentive to develop computational methods to detect potential OMIM disease-miRNA associations. In this paper, random walk on OMIM disease similarity network is applied to predict potential OMIM disease-miRNA associations under the assumption that functionally related miRNAs are often associated with phenotypically similar diseases. Our method makes full use of global disease similarity values. We tested our method on 1226 known OMIM disease-miRNA associations in the framework of leave-one-out cross-validation and achieved an area under the ROC curve of 71.42%. Excellent performance enables us to predict a number of new potential OMIM disease-miRNA associations and the newly predicted associations are publicly released to facilitate future studies. Some predicted associations with high ranks were manually checked and were confirmed from the publicly available databases, which was a strong evidence for the practical relevance of our method.

## 1. Introduction

MicroRNAs (miRNAs) are a class of small noncoding RNAs typically about 22 nucleotides in length. They have been identified in eukaryotic organisms ranging from nematodes to humans [1–3]. *Caenorhabditis elegans *(*C. elegans*) lin-4 and let-7 are the first two discovered miRNAs [4, 5]. Over the past decade, thousands of miRNAs have been discovered. miRNAs normally function as negative regulators of gene expression [6–8]. Research also reports that miRNAs may act as positive regulators in some cases [9, 10].

Many investigators have reported that miRNAs are critical in tissue development [11], cell growth [12], cellular signalling [13], and so on. As such, the mutation of miRNAs, the dysfunction of miRNA biogenesis, and the dysregulation of miRNAs and their targets may result in various diseases, such as lung cancer [14], lymphoma [15], and breast cancer [16]. Therefore, research on the relationship between miRNAs and diseases has become an important biomedical goal.

The Online Mendelian Inheritance in Man database (OMIM, http://www.ncbi.nlm.nih.gov/omim/) is a comprehensive knowledgebase of human genetic disorders. It contains information about genes and genetic phenotypes. As of December 2012, OMIM comprised 5442 Mendelian diseases (which are prefixed using “#” (*n* = 3676) if the responsible gene is known and with “%” otherwise (*n* = 1766)). However, efforts made to reveal OMIM disease-miRNA associations are lacking and the majority of diseases in the OMIM database are not associated with any miRNA. To provide testable hypotheses to guide future experiments, it is of great importance to devise computational models to infer potential OMIM disease-miRNA associations.

Recently, some important conclusions and computational methods about the relationship between diseases and miRNAs have been presented. Lu et al. [17] performed a comprehensive analysis to the human miRNA-disease association data and disclosed that miRNAs tend to show similar or different dysfunctional evidences for the similar or different disease clusters, respectively. Jiang et al. [18] proposed a computational model based on the hypergeometric distribution to infer potential miRNA-OMIM disease associations by prioritizing the entire human microRNAome for diseases of interest. The notation that functionally related miRNAs tend to be associated with phenotypically similar diseases was reconfirmed in their manuscript. Although miRNAs functional network, disease similarity network, and known miRNA-disease associations were integrated in their work, only the neighbor information of each miRNA was used in their scoring system. Prediction accuracy would be increased by taking advantage of the global network similarity information. Another limitation is that *in silico* predicted associations were used as data sources in this method. It is known that these predicted associations used as data sources have some false-positive and false-negative results, thus influencing the final prediction accuracy. To test the hypothesis that some miRNAs could be potentially responsible for a number of “orphan” OMIM diseases, Rossi et al. [19] developed a novel approach OMiR to calculate the significance of the overlap between miRNA loci and OMIM disease loci. Results suggested that “orphan” genetic disease loci were proximal to miRNA loci more frequently than to loci for which the responsible protein coding gene is known.

In this paper, we propose a computational approach to infer potential human OMIM disease-miRNA associations by random walk to prioritize the candidate diseases for miRNAs of interest. We first constructed an OMIM disease similarity network and an OMIM disease-miRNA association network. We subsequently implement random walk on the OMIM disease similarity network to prioritize candidate diseases for an miRNA of interest. Cross-validation has illustrated the excellent performance of our method. The comprehensively predicted OMIM disease-miRNA associations also enable us to suggest many potential OMIM disease-miRNA associations, which can offer help in further experiments and hence increase research productivity. We further manually checked some strongly predicted associations and encouraging confirmation results were found from the publicly available databases.

## 2. Materials and Methods

### 2.1. Data Preparation

The benchmark dataset (see Supplementary material S1 available at http://dx.doi.org/10.1155/2013/204658) used in this manuscript is downloaded from [20, 21]. Here below we provide a brief description.

#### 2.1.1. The OMIM Disease Similarity Data

We download the disease phenotype similarity scores from the MimMiner [21], developed by van Driel et al. who computed a phenotype similarity score for each phenotype pair by the text mining analysis of their phenotype descriptions in the Online Mendelian Inheritance in Man (OMIM) database [22]. The phenotypic similarity scores have been successfully used to predict or prioritize disease-related protein-coding genes [23, 24].

OMIM disease similarity matrix is defined as *O*, where the entity *O*(*i*, *j*) in row *i* column *j* is the similarity score between OMIM disease *i* and *j*. Based on the similarity matrix, OMIM disease similarity network (ODSN) is constructed, where vertex set *D* = {*d*_1_, *d*_2_,…, *d*_*n*_} denotes the set of *n* OMIM diseases. Vertices *d*_*i*_ and *d*_*j*_ are linked by an edge in the network if the similarity between diseases *i* and *j* is more than zero. The similarity score between diseases *i* and *j* is used as the weight of this edge.

#### 2.1.2. The OMIM Disease-miRNA Association Data

Previous studies have produced a large number of miRNA-disease associations. Lu et al. [17] and Jiang et al. [20] manually retrieved the associations of miRNA and disease from literatures and constructed two curated databases, human miRNA-associated disease database (HMDD) and miR2Disease, respectively. They aim to offer comprehensive resources of experimentally confirmed miRNA-disease associations. Yang et al. [25] also created a publicly available database of Differentially Expressed miRNAs in human Cancers (dbDEMC) with the goal to provide potential cancer-related miRNAs by *in silico* computing.

The OMIM disease-miRNA association data used in our paper was downloaded from miR2Disease [20]. After mapping these downloaded diseases into OMIM disease IDs, we finally received 1226 OMIM disease-miRNA associations consisting of 61 OMIM diseases and 365 miRNAs. These associations were used for performance evaluation, and the latest versions of the HMDD [17] and dbDEMC [25] data were applied for prediction confirmation.

OMIM disease-miRNA association network (ODMAN) was constructed based on the 1226 verified associations, where vertex set *D* = {*d*_1_, *d*_2_,…, *d*_*n*_} denotes the set of *n* OMIM diseases and *M* = {*m*_1_, *m*_2_,…, *m*_*k*_} denotes the set of *k*miRNAs. Vertices *m*_*i*_ and *d*_*j*_ are linked by an edge in the ODMAN if disease *j* is associated with miRNA *i* in our datasets. The weights of all edges are set to be 1.

### 2.2. Method Description

Random walk is a ranking algorithm. It simulates a random walker who starts on some given seed nodes and moves to their immediate neighbors randomly at each step. Finally, all the nodes in the network are ranked by the probability of the random walker reaching this node. Let *p*_0_ be the initial probability vector and *p*_*s*_ a vector in which the *i*th element holds the probability of finding the random walker at node *i* at step*s*. The probability vector at step *s* + 1 can be given by

(1) $$\begin{array}{c} p_{s+1}=rp_{0}+\left(1-r\right)D_{\mathrm{norm}}p_{s}, \end{array}$$

where *r* is the restart probability of random walk in every time step at source nodes and *D*_norm⁡_ is the normalized similarity network.

After some steps, the probability will reach a steady state. This is obtained by performing the iteration until the difference between *p*_*s*_ and *p*_*s*+1_ (measured by the *L*1 norm) falls below 10^−10^. The steady-state probability *p*_*∞*_ gives a measure of proximity to seed nodes. If *p*_*∞*_(*i*) > *p*_*∞*_(*j*), then node *i* is more proximate to seed nodes than node *j*.

In this paper, based on the observation that functionally related miRNAs are often associated with phenotypically similar diseases [17], random walk was proposed to uncover the potential associations between OMIM diseases and miRNAs. The source code in Matlab can be downloaded from Supplementary Material S2. As we want to predict potential OMIM diseases for a given miRNA *m* of interest, all the OMIM diseases which have already been confirmed to be associated with this miRNA will be considered as seed nodes. Other nonseed OMIM diseases will be considered as candidate diseases. The initial probability *p*_0_ is formed such that equal probabilities are assigned to the seed nodes, with the sum equal to 1, while the initial probabilities of nonseed miRNAs are 0. Here we allow the restart of random walk in every time step at source nodes with probability *r*  (0 < *r* < 1). After some iteration, the random walk is stable. The stable probability is defined as *p*_*∞*_. Candidate OMIM diseases are ranked according to *p*_*∞*_. The high-scored OMIM diseases can be expected to have a high probability to be associated with the given miRNA.

## 3. Results

### 3.1. OMIM Disease-miRNA Association Network (ODMAN) Analysis

**Figure 1:**
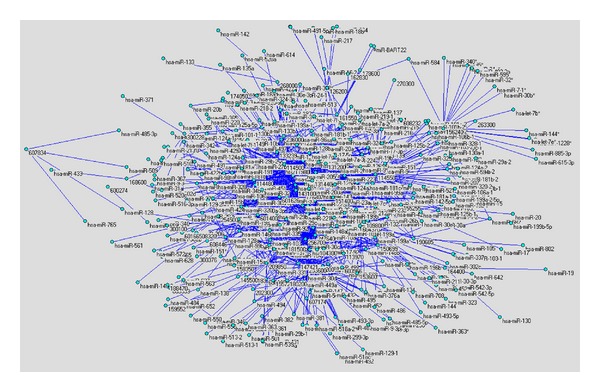
OMIM disease-miRNA association network (ODMAN). The network is generated by using 1226 experimentally verified associations between OMIM diseases and miRNAs. The network is prepared by Pajek (http://vlado.fmf.uni-lj.si/pub/networks/pajek/).

In this study, we first focus on the verified OMIM disease-miRNA associations. The set of 1226 known OMIM disease-miRNA associations is regarded as the “gold standard” data and is used for evaluating the performance of our proposed method in the cross-validation experiments as well as training data in the comprehensive prediction. We constructed the OMIM disease-miRNA association network using a bipartite graph representation (see Figure 1) and analyzed some statistics for the OMIM disease-miRNA association network. In the bipartite graph, the heterogeneous nodes correspond to either miRNAs or diseases, and edges correspond to associations between them. An edge is placed between a miRNA node and a disease node if the disease is known to associate with the miRNA.

**Figure 2:**
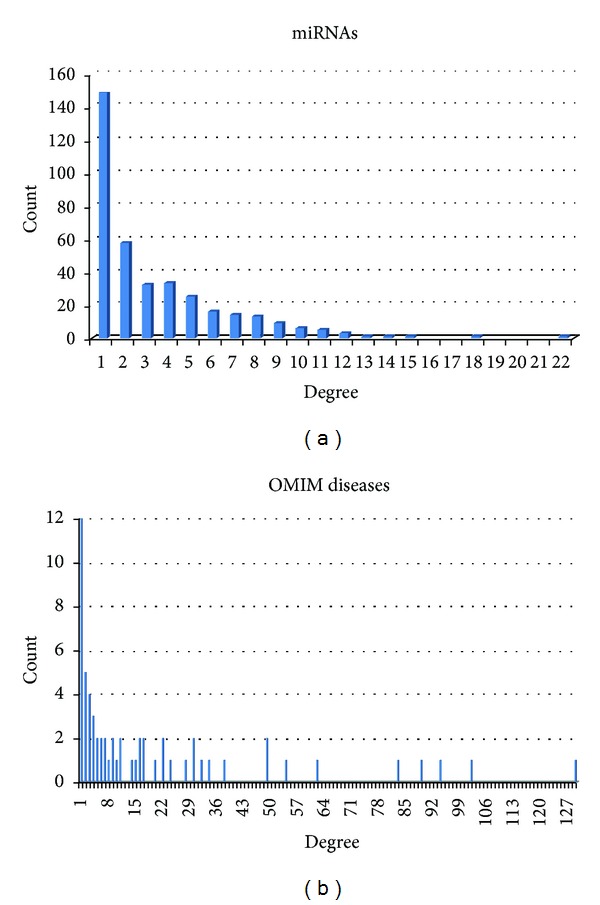
Degree distributions for OMIM diseases and miRNAs in the OMIM disease-miRNA association network (ODMAN). (a) shows the histograms of the degree of miRNAs. (b) shows the histograms of the degree of OMIM diseases.

Figure 2 shows the degree distributions for miRNAs and diseases in the OMIM disease-miRNA association network. The degree of the miRNA (respective disease) node is the number of diseases that the miRNA has associations with (resp., the number of miRNAs targeting the disease).

Table 1 details some statistics for the OMIM disease-miRNA association network, such as the average degree of miRNAs and the average degree of diseases.

Inspection of the OMIM disease-miRNA association network shows that most edges in the network are connected and form a large connecting subnetwork.

### 3.2. Performance Evaluation of the Proposed Method

In order to assess the power of our method to predict OMIM disease-miRNA associations by prioritizing the entire candidate OMIM diseases, we performed a leave-one-out cross-validation on the 1226 known OMIM disease-miRNA associations. For a given miRNA *m*, each known related OMIM disease was left out in turn as test disease and other known OMIM diseases were taken as seed nodes. The candidate disease set consisted of all the OMIM diseases which have no evidence to show their association with miRNA *m*.

We calculated the sensitivity and specificity for each threshold. Sensitivity refers to the percentage of the associations whose ranking is higher than a given threshold, namely, the ratio of the successfully predicted experimentally verified OMIM disease-miRNA associations to the total experimentally verified OMIM disease-miRNA associations. Specificity refers to the percentage of associations that are below the threshold. The value of area under receiver-operating characteristics (ROC) curve (AUC) was calculated and an AUC value of 71.42% was achieved, suggesting that our method can recover the known experimentally verified OMIM disease-miRNA associations and therefore has the potential to infer new OMIM disease-miRNA associations.

### 3.3. Effects of Parameter in the Proposed Method

Restart probability *r* is one parameter in our method. To investigate the selection of the parameter for the performance of our method, we set various values for it and calculated the AUC values in the framework of leave-one-out cross-validation.  Table 2 details the effect of the parameter on the cross-validation results in the benchmark dataset. After a comprehensive searching, the parameter (*r* = 0.8) which led to best AUC result is selected for further association prediction. It could also be observed that the predictive result is robust to the restart probability.

### 3.4. Comparison with Other Methods

Until recently, efforts made to discover potential OMIM disease-miRNA associations are lacking. Meanwhile models have been constructed based on different data features, which makes direct performance comparison difficult. The most recent study related with our work is the computational model proposed by Jiang et al. [18], which was based on the hypergeometric distribution to infer potential miRNA-OMIM disease associations by prioritizing the entire human microRNAome for diseases of interest. Only the neighbor information of each miRNA was used in their scoring system. Another limitation is that *in silico* predicted associations were used as data sources in this method. It is known that these predicted associations have some false-positive and false-negative results, which may bring noises to the experiments. Our method is based on the experimentally verified OMIM disease-miRNA associations.

### 3.5. Comprehensive Prediction for Unknown OMIM Disease–miRNA Associations

After confirming the usefulness of our method, we conduct a comprehensive prediction of unknown associations between all possible OMIM diseases and miRNAs. In the inference process, we trained our method with all the known associations. We ranked the nonassociating pairs with respect to their probability scores and extracted the top 20 predicted associations for each of the 365 OMIM diseases. The full list of the prediction results can be obtained from Supplementary Material S3.

Furthermore, we manually checked some strongly predicted associations. Take the top 10 predicted associations of *hsa-let-7g* as an example. We confirmed that 6 associations (Table 3) are now annotated in at least one of the two latest online versions of HMDD [17] and dbDEMC [25] databases. We take these as a strong evidence to support the practical application of our method. Note that the predicted associations that are not reported yet may also exist in reality.

## 4. Discussion

We have applied random walk on OMIM disease similarity network to predict potential OMIM disease-miRNA associations. Differing from using local network similarity measures, like the method proposed by Jiang et al. [18], we adopted global network similarity measures. Excellent performance based on leave-one-out cross-validation suggested that our method has the potential to infer new OMIM disease-miRNA associations. The newly predicted associations are publicly released to facilitate future studies. Further confirmation of some strongly predicted associations in publicly accessible databases indicates the realistic application of our method.

Despite the encouraging results of our method, there are also limitations. The known experimentally verified OMIM disease-miRNA associations were rare. Therefore, integrating other bioinformatics sources, such as Gene Ontology, might improve model performance. Our method cannot be applied for miRNAs which do not have any known associated OMIM diseases. Thus miRNA similarity information should be taken into consideration. From a technical viewpoint, the performance of our method could be improved by using more accurate similarity information designed for OMIM diseases.

## Supplementary Material

Supplementary Material S1: The benchmark dataset used in this manuscript.
Supplementary Material S2: The source code used in this manuscript.
Supplementary Material S3: The full list of prediction results.

## Figures and Tables

**Table 1:** Statistics for the OMIM disease-miRNA association network.

No. of OMIM diseases	No. of miRNAs	No. of OMIM disease-miRNA associations	Average degree of OMIM diseases	Average degree of miRNAs
61	365	1226	20.10	3.36

**Table 2:** The effect of restart probability value on the cross-validation results.

Restart probability	0.1	0.2	0.3	0.4	0.5	0.6	0.7	0.8	0.9
AUC	0.6703	0.6903	0.7011	0.7082	0.7126	0.7135	0.7138	0.7142	0.7138

**Table 3:** The newly confirmed OMIM disease-miRNA associations in the top 10 predicted results of *hsa-let-7g*.

miRNA	OMIM ID	Rank	Source
*hsa-let-7g*	114480	1	HMDD
*hsa-let-7g*	155720	2	HMDD
*hsa-let-7g*	180200	3	HMDD
*hsa-let-7g*	256700	4	
*hsa-let-7g*	188470	5	
*hsa-let-7g*	109800	6	
*hsa-let-7g*	133239	7	dbDEMC
*hsa-let-7g*	603956	8	HMDD
*hsa-let-7g*	155255	9	dbDEMC
*hsa-let-7g*	607174	10	
